# Comparison of the approaches to combine Renal Replacement Therapy with Extracorporeal Membrane Oxygenation – a Scoping Review

**DOI:** 10.1016/j.aicoj.2026.100165

**Published:** 2026-09-21

**Authors:** Eden Kharrouba, Quentin Moyon, Matthew E. Cove, Jutta Arens, Ana Martins Costa, John F. Fraser, Jacky Y. Suen

**Affiliations:** a The University of Queensland, Institute for Molecular Bioscience, Brisbane, Australia; b Critical Care Research Group, The Prince Charles Hospital, Brisbane, Australia; c Service de Médecine Intensive Reanimation, Hôpital de la Pitié-Salpêtrière, Sorbonne Université, Paris, France; d Department of Medicine, National University Hospital, Singapore, Singapore; e Engineering Organ Support Technologies Group, Department of Biomechanical Engineering, University of Twente, Enschede, the Netherlands; f School of Medicine, Griffith University, Brisbane, QLD, Australia

**Keywords:** Acute kidney injury, Renal replacement therapy, Extracorporeal membrane oxygenation, Combination, Integration

## Abstract

**Introduction:**

Extracorporeal membrane oxygenation (ECMO) is increasingly used for cardiac and respiratory failure and is associated with high rates of acute kidney injury (AKI) and fluid overload requiring renal replacement therapy (RRT). ECMO and RRT can be run independently in parallel, or RRT hemofilter or device can be integrated into the ECMO circuit. Several parallel and integrated ECMO-RRT configurations have been described, but the optimal approach remains uncertain.

This scoping review mapped the available literature comparing at least two ECMO-RRT configurations, summarized reported outcomes, and identified knowledge gaps.

**Methods:**

We searched PubMed, Cochrane Library, Web of Science, Embase, and ClinicalTrials.gov without restrictions, with the final search conducted on February 9, 2026. Eligible studies compared at least 2 ECMO-RRT configurations linked to reported outcomes. Data on study characteristics, configurations, outcomes, and anticoagulation were extracted and synthesized.

**Results:**

Of 6,806 records, 20 studies were included: 12 were clinical including 1 randomized controlled trial, 3 in vitro, 1 in silico, and 4 surveys. More than 8 ECMO-RRT connection schemes have been described by clinical studies since 2013. No consistent differences in efficiency or clinical outcomes were observed between the configurations compared. Integrated methods were associated with longer or comparable hemofilter lifespan. Surveys confirmed heterogeneous medical practices, and 2 safety concerns (unstoppable pressure alarms and air embolism) were reported.

**Conclusion:**

Evidence on ECMO-RRT configurations is limited and heterogeneous. Future systematic and rigorous comparison of all the configurations through preclinical and clinical studies is needed to harmonize medical practices and inform the development of guidelines.

## Introduction

Extracorporeal Membrane Oxygenation (ECMO) is indicated in cases of refractory cardiac or respiratory failure. Veno-arterial (V-A) ECMO is provided in the context of cardiogenic shock, while veno-venous (V-V) ECMO supports lung function for patients suffering from pulmonary failure. International data from the Extracorporeal Life Support Organization describes a mortality rate higher than 50% for patients treated with V-A ECMO, and 40% for V-V ECMO [1]. Over half of V-V and V-A ECMO patients have concomitant acute kidney injury (AKI) and require renal replacement therapy (RRT), associated with increased mortality risk [[2], [3], [4]].

Various ECMO-RRT connection configurations exist and can be divided into parallel and integrated approaches. The parallel approach consists of running ECMO and RRT using separate vascular access, while integrated approaches connect the RRT circuit directly to the ECMO circuit. The parallel approach is considered advantageous because the devices do not interfere with one another. However, additional vascular access is not always available and creates additional risk for cannula infection, bleeding, and thrombotic events, potentially increasing the mortality rate of these patients [5,6]. While integrated approaches reduce the number of cannula access sites required, they face technical challenges related to the differential pressure and flow ranges of RRT and ECMO. High ECMO circuit pressures can trigger CRRT pressure alarms, resulting in treatment interruptions and making it difficult to ensure that the targeted therapeutic dose is accurately delivered to the patient [5,6].

Therefore, it remains unclear which approach is best in terms of safety, quality of therapy delivery, and feasibility. Consequently, current guidelines cannot recommend a specific approach, and medical practice is based on hospital experience rather than scientific evidence [7]. A recent review has focused primarily on the technical and practical considerations of ECMO-CRRT integration [8]. In contrast, this scoping review seeks to map the breadth and nature of the available evidence regarding ECMO-RRT configurations by identifying and characterizing studies comparing at least two ECMO-RRT configurations, critically appraising their methodological quality, and summarizing both clinical outcomes and technical performance. This review also aimed to highlight knowledge gaps and inform future research and evidence-based guideline development.

## Methods

### Scoping review

This review was conducted as a scoping review, aligned with the JBI Manual for Evidence Synthesis and the PRISMA extension for Scoping Reviews (PRISMA-ScR) [9,10]. The PRISMA-ScR checklist is available as the additional file 1. Although the research question addressed could ultimately be answered through a systematic review, a scoping review was considered the most appropriate design to systematically map and characterize the extent and nature of the available evidence, identify knowledge gaps and research priorities, and provide a foundation for future research and, where appropriate, subsequent systematic reviews as the evidence base matures.

### Protocol and registration

Our protocol was registered in Open Science Framework on the 22nd of October 2025 [11].

### Search strategy

Sources of evidence were retrieved from PubMed, Cochrane Library, Web of Science, Embase, and Clinicaltrials.gov, using queries described in additional file 2. A first search was performed on the 20th of July 2025, with no restrictions on source type, date, population, or study design, including in vitro studies (physical experiments using extracorporeal circuits or loops), in silico studies (computational modelling or simulation), and clinical studies involving patients. A subsequent search (9th of February 2026) was carried out prior to manuscript submission to ensure the most recent evidence was captured.

All the references were managed using Zotero. After combining the results from all the databases, duplicates were removed using the Deduplicator tool in Systematic Review Accelerator [12]. The deduplicated results were imported into Covidence for screening.

### Eligibility criteria

Studies were included if they compared at least two approaches to simultaneously provide ECMO and RRT to patients. Surveys and case reports that included at least 4 cases were included. Studies were excluded if they reported only one configuration or did not link the configurations to any described outcome. Reviews, letters to the editor, and editorials were excluded to ensure we investigated insights from original and peer-reviewed articles only. For the same reason, non-peer-reviewed sources, such as conference abstracts, posters, and preprint manuscripts, were excluded. Articles written in languages other than English were excluded.

### Selection of studies

Two reviewers (EK and QM) independently performed title and abstract screening. Discrepancies were handled through discussion until a consensus was reached. Full text screening was performed the same way.

### Data charting and items

Data charting was conducted by retrieving information from the main text and supplementary documents. Characteristics of the study included the study type and design, country, the population of interest (adults or pediatrics) and the number of patients, their dates of enrolment, the type of ECMO and RRT, as well as the configurations used to combine them and the type of outcomes reported by the study. The outcomes observed in each study and the related conclusions were extracted, including their quantifications as stated in the original publications. The details of anticoagulation strategies were retrieved, within the limit of their description in the included studies.

### Synthesis of results

All data extracted from the studies were summarized in tables or graphical representations. Qualitative reporting of observations from each study was performed for each outcome type.

### Critical appraisal

A critical appraisal analysis was conducted to assess the quality of the clinical studies using the JBI critical appraisal tool for clinical studies and randomized controlled trials [13,14] and reported as additional files 4 and 5.

## Results

### Selection of sources of evidence

The initial search retrieved 6,806 references, among which 2,293 were duplicates and were removed prior to the title and abstract screening. After the title and abstract screening phase, 301 studies were selected for full-text retrieval. 282 studies were excluded due to incorrect article type (preprints or not peer-reviewed), or inadequate setting and reporting (Fig. 1). One additional reference published after our initial search was found during our final search in 2026 and included. Therefore, 20 references were included in this scoping review.

**Fig. 1:**
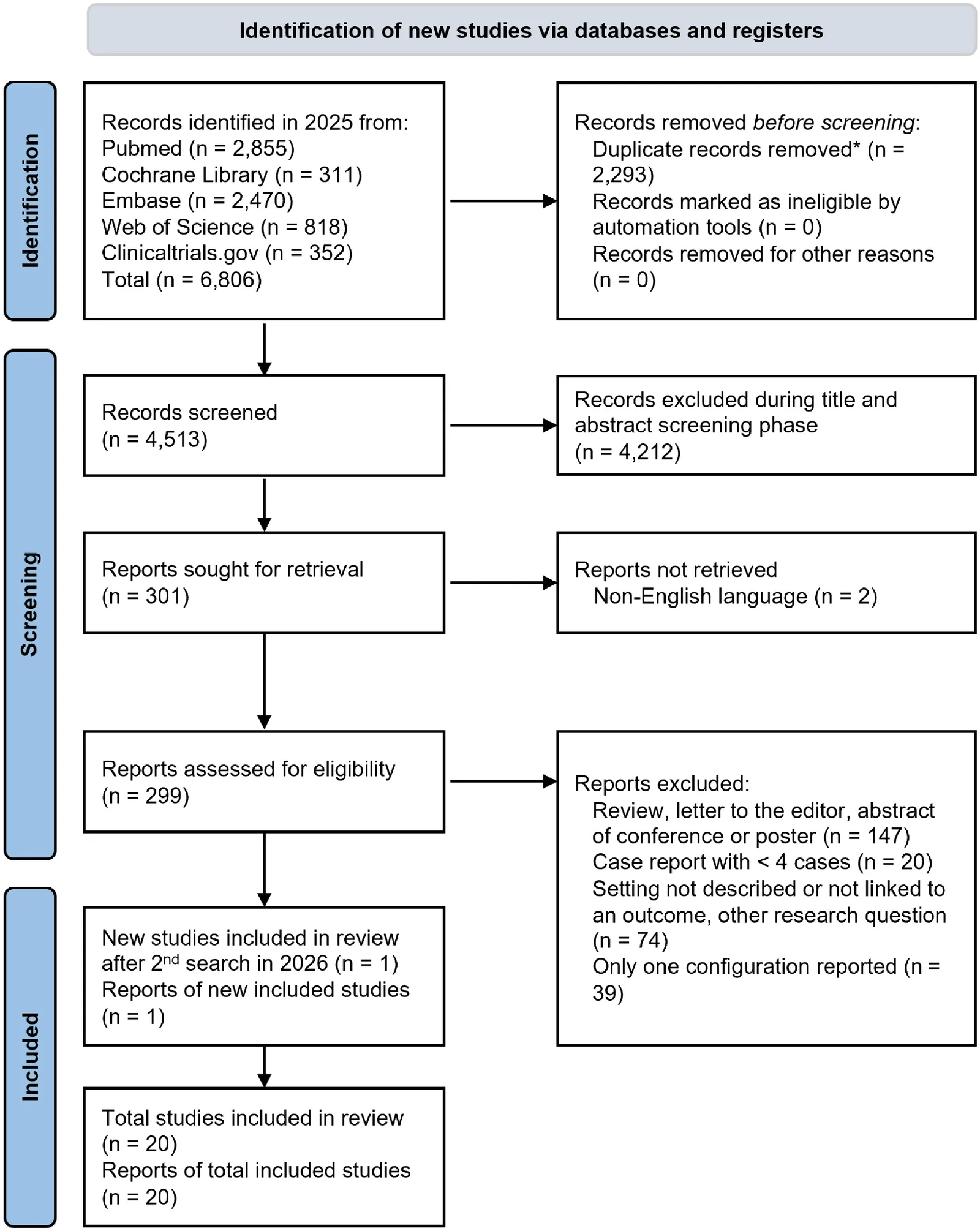
PRISMA flow diagram. Legend: PRISMA flow diagram describing the inclusion process. * Deduplication was performed both manually and using Systematic Research Accelerator Deduplicator tool.

### Characteristics of the included studies

The characteristics of the included studies are presented in Table 1; 12 studies were clinical [[15], [16], [17], [18], [19], [20], [21], [22], [23], [24], [25], [26]], 3 in-vitro [[27], [28], [29]], 1 in-silico [30] (Table 1a), and the 4 remaining were surveys on medical practices [[31], [32], [33], [34]] (Table 1b).

**Table 1:** Characteristics of the 20 studies included in the scoping review.

Table 1a				
Study	Country	Study type	Population	Aim of the study
Symons et al. (2013)	USA	Clinical, retrospective, single center	Pediatrics (n = 42); V-A/V-V: NA; In-line hemofilter post-pump→prepump (n = 30) vs. integrated RRT device post-pump→pre-oxygenator or post-pump→prepump (n = 12)	Compare CRRT and free flow hemofilter regarding ultrafiltration precision
De Tymowski et al. (2017)	France	Clinical, single center	Adults (n = 12); V-A (n = 8); V-V (n = 4); Integrated RRT device (n = 12); sequential: post-pump→pre-oxygenator, pre-pump→post-pump, pre-pump→pre-pump	Proposed a standardized protocol for the management of pressure-triggered alarms
Na et al. (2018)	South Korea	Clinical, single center	Adults (n = 170); V-A (n = 127); V-V (n = 40); Mixed (n = 3); Integrated RRT device post-pump→prepump with pressure lines: 0 (n = 16), 1 (n = 36), 2 (n = 118)	Prove the feasibility in terms of safety and efficiency of managing pressures in the RRT circuit
Gorga et al. (2020)	North America	Clinical, retrospective observational, multicentric	Pediatrics (n = 357); V-A (n = 265); V-V (n = 92); Integrated RRT device (n = 81) vs. in-line hemofilter (n = 276) connections not described	Differences in in-hospital mortality between CRRT and hemofilter are described
Worku et al. (2021)	USA	Clinical, retrospective, single center	Adults (n = 25); V-A; Parallel (n = 22 runs) vs. integrated RRT device post-pump→preoxygenator (n = 22 runs)	Feasibility of the integrated approach in terms of safety and efficiency
Liu et al. (2021)	China	Clinical, single center	Adults (n = 21); V-A (n = 16); V-V (n = 3); V-AV (n = 2); Integrated RRT device post-pump→prepump (n = 8) vs. post-oxygenator→preoxygenator (n = 13)	Assess the differences between two integrated approaches regarding safety and efficiency
Wu et al. (2023)	China	Clinical, retrospective observational, single center	Adults (n = 100); V-A (n = 70); V-V (n = 30); Parallel (n = 30) vs. integrated RRT device post-oxygenator→preoxygenator (n = 45) or post-oxygenator→prepump (n = 25)	Feasibility and advantages of the integrated approach in terms of safety and efficiency
Raja et al. (2023)	United Kingdom	Clinical, single center, prospective	Adults (n = 20); V-V; Parallel (n = 10) vs. integrated RRT device post-oxygenator→preoxygenator (n = 10)	Report on medical practices and management of the patients between integrated and parallel approaches
Ciullo, Knecht et al. (2023)	USA	Clinical, retrospective observational, single center	Adults (n = 33); Type of ECMO: NA; Parallel (n = 26) vs. integrated RRT device (n = 7, connection not described)	Feasibility and advantages of integrated approach compared to parallel one regarding safety and efficiency
Huang et al. (2023)	China	Clinical, single center	Adults (n = 120); V-A (n = 60); V-V (n = 60); Integrated RRT device (n = 120); pre-oxygenator→postoxygenator with additional pressure controls (n = 60) vs. without (n = 60)	Benefit of additional pressure control lines
Schönfelder et al. (2025)	Germany	Clinical, retrospective observational, single center	Adults (n = 105); V-V; Parallel (n = 48) vs. integrated RRT device post-oxygenator→preoxygenator (n = 57)	Comparison of integrated vs. parallel approach in terms of safety and efficiency
Tangjitaree et al. (2026)	Thailand	Clinical, randomized controlled trial, multicenter	Adults (n = 80); V-A (n = 67); V-V (n = 13); Parallel (n = 40) vs. integrated RRT device post-pump→preoxygenator (n = 40)	Comparison of integrated vs. parallel approach in terms of CRRT filter lifespan
Suga et al. (2017)	Japan	In-vitro	V-V and V-A; Integrated RRT device post-pump→pre-pump (V-V) and post-oxygenator→pre-pump (V-A)	Technical and hemodynamic considerations
Profeta et al. (2018)	USA	In-vitro	Neonatal ECMO circuit V-A (Centrifugal or roller pump with Quadrox-ID pediatric oxygenator); Integrated RRT device; 7 integrated configurations	Technical and hemodynamic considerations
Shank et al. (2018)	USA	In-vitro	Neonate; V-A; Integrated RRT device; post-pump→pre-oxygenator, pre-pump→post-pump, post-pump→pre-pump	Technical and hemodynamic considerations and micro-emboli
Thiel et al. (2025)	Germany	In-silico	Adults (n = 8); V-A; Integrated RRT device; 9 integrated configurations	Technical and hemodynamic considerations

Table 1b						
Survey	Total number of respondents	Respondents Specialty	Respondents’ country	ECMO modes reported	RRT modes reported	Configurations reported
Fleming et al. (2012)	65	Pediatric and adult centers (n = 24), Pediatric only (n = 37) and adult only (n = 2)	International - estimations USA: 52 (80%) Canada: 3 (5%) Europe: 7 (10%) Australia or New Zealand: 3 (5%)	Respiratory or cardiac indication of ECMO	SCUF, CVVH, CVVHD, CVVHDF	In-line hemofilter, CRRT integrated (pre-pump, post-pump to pre-oxygenator and other not described configurations)
Bidar et al. (2021)	90	Intensivists (n = 90) including pediatric specialists (n = 20)	France and Switzerland	V-V or V-A	IHD, CVVH, CVVHD, CVVHDF	In-line hemofilter or CRRT, pre-pump, pre-pump to post-pump, post-oxygenator to pre-oxygenator
Gorga et al. (2022)	60	Neonatal and pediatric specialists only (n = 25), neonatal, pediatric and adults (n = 35)	International – no details	Respiratory or cardiac indication of ECMO	SCUF, CVVH, CVVHD, CVVHDF, SLED	In-line hemofilter, CRRT integrated or parallel – connection not described
Thy et al. (2022)	298	Medical intensivist (n = 101), anesthesiologist-intensivist (n = 97), medical physician (n = 12), Pediatrician (n = 34), surgeon (n = 32)	International Europe: 141 (47%) North America: 87 (29%) Asia: 35 (12%) South America: 23 (8%) Oceania: 7 (2%) Africa: 2 (1%)	V-V or V-A	IHD, CVVH, CVVHD, CVVHDF	Parallel vs. integrated Description of the access and return cannula positions for the integrated approaches without describing an entire couple of inlet and outlet positions

Legend: Characteristics of the 20 studies included in the scoping review in terms of type, country, population of patients or respondents, settings and outcomes considered. Table (1a) present the preclinical and clinical studies while Table (1b) relates to the survey on medical practices. Patients were adults if older than 18 years old (age ≥18 years old). Studies are organized by type (clinical, in-vitro, in-silico surveys) and in chronological order.

Among the 12 clinical studies, the earliest was published in 2013 [15] and enrolled patients from 2005 to 2010, while the most recent was published in 2026 [24] and recruited patients from May 2021 to March 2025. Only two studies were multicentric, including a randomized controlled trial [18,24], and 2 were conducted with pediatric patients [15,18]. The patient pool in the adult population ranged from 12 to 170 patients, with different ECMO modes provided in 7 studies [[16], [17], [18],[22], [23], [24], [25]], V-A ECMO only in 1 study [19], and V-V ECMO only in 2 others [20,26].

Among the studies reporting the RRT mode, continuous renal replacement therapy (CRRT) and in-line hemofiltration were common, with both continuous veno-venous hemodialysis (CVVHD) and continuous veno-venous hemodiafiltration (CVVHDF) being reported in 4 cases [16,17,19,24,[26], [27], [28]].

Fig. 2 summarizes the main outcomes assessed in each study and the configurations compared. The 4 in-vitro and in-silico studies analyzed the technical parameters of integrating ECMO and RRT, such as pressures and flows in the two circuits across multiple integrated connection schemes. Three clinical studies also focused on managing pressures within the CRRT circuit across different integration approaches [16,17,23]. Two studies compared integrated CRRT and in-line hemofilter, assessing precision of ultrafiltration [15] and mortality rates [18]. Finally, among the other 7 clinical studies focused on patients’ outcomes, 6 compared the parallel approach to at least one integrated one [[19], [20], [21], [22],^24^,^26^], and 1 compared two integrated approaches [25].

**Fig. 2:**
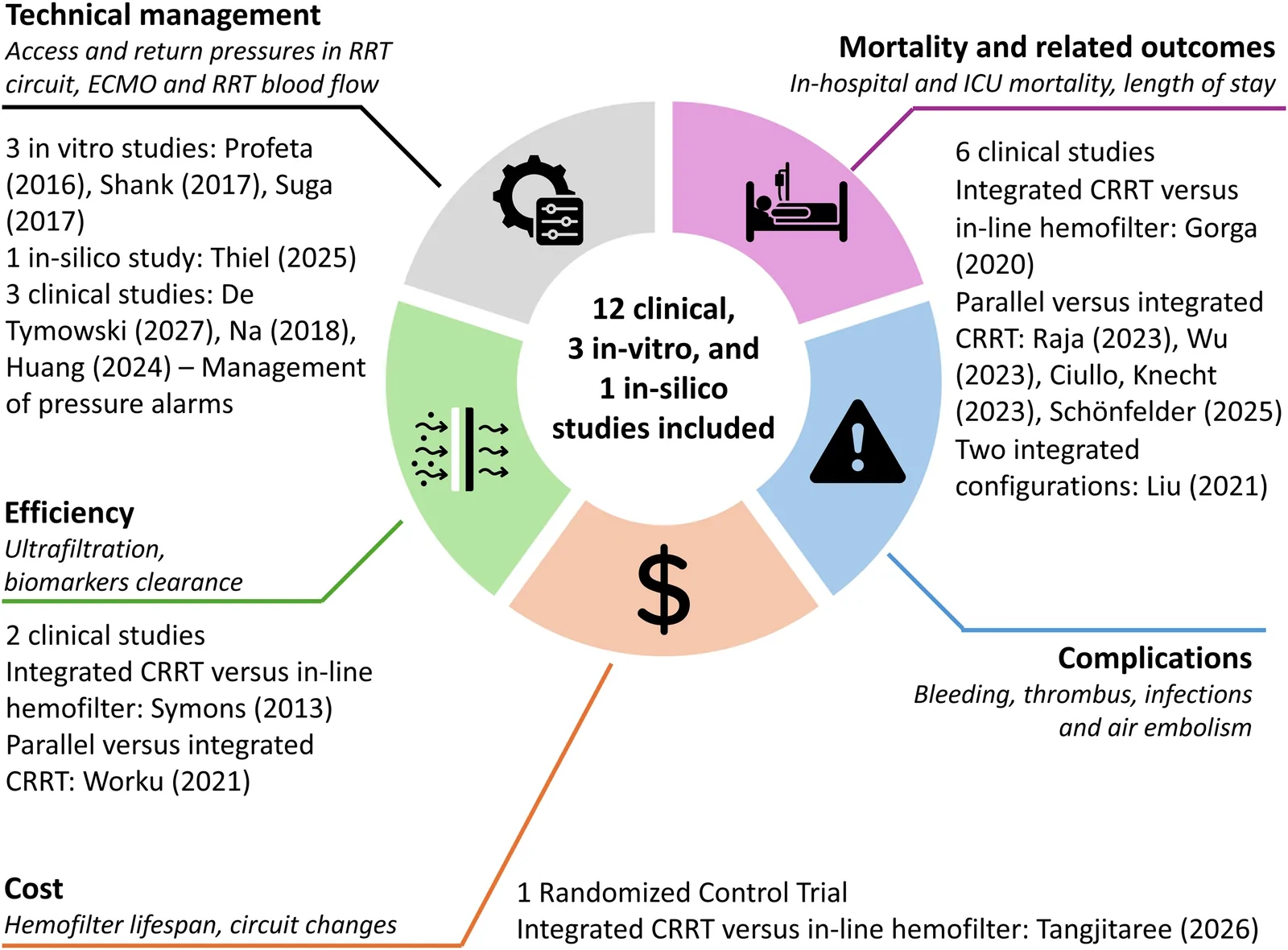
Main types of criteria considered in the assessment of RRT-ECMO integration. Legend: Visual representation of the main criteria used in the 16 clinical, in-vitro, and in-silico studies included to compare different configurations between ECMO and RRT. For each criterion, the list of the studies for which it was the main outcome is written in italic below (and above for the “complications” category), alongside the treatments compared.

All the surveys were multicenter international surveys, with physician respondents, including pediatric specialists. Two surveys, Fleming et al. (2012) and Gorga et al. (2022) had a higher representation of pediatric specialists [[31], [32], [33], [34]].

### Description of the configurations

Various configurations have been described in the included studies to combine ECMO and RRT, as detailed in Table 1 and represented in Fig. 3. Among the clinical studies, parallel approach was the most mentioned (n = 6 studies and 179 patients pooled) [[19], [20], [21], [22],^24^,^26^], and the most commonly described integrated and in-line hemofilter configurations were; (1) post-oxygenator to pre-oxygenator (n = 4 studies, 125 patients) [20,22,25,26], (2) post-pump to pre-oxygenator (n = 4 studies, 74 patients, including one study using an in-line hemofilter) [15,16,19,21,24], (3) post-pump to pre-pump (n = 3 studies, 54 patients pooled) [15,17,25]. Less common configurations included pre-pump to post-pump [16], post-oxygenator to pre-pump [22], and pre-pump to pre-pump [16]. Interestingly, one clinical study reported the integration of CRRT from pre-oxygenator to post-oxygenator, bypassing the oxygenator [23]. In-silico and in-vitro studies extended the configuration possibilities with return line of the RRT circuit connected post-oxygenator [29,30].

**Fig. 3:**
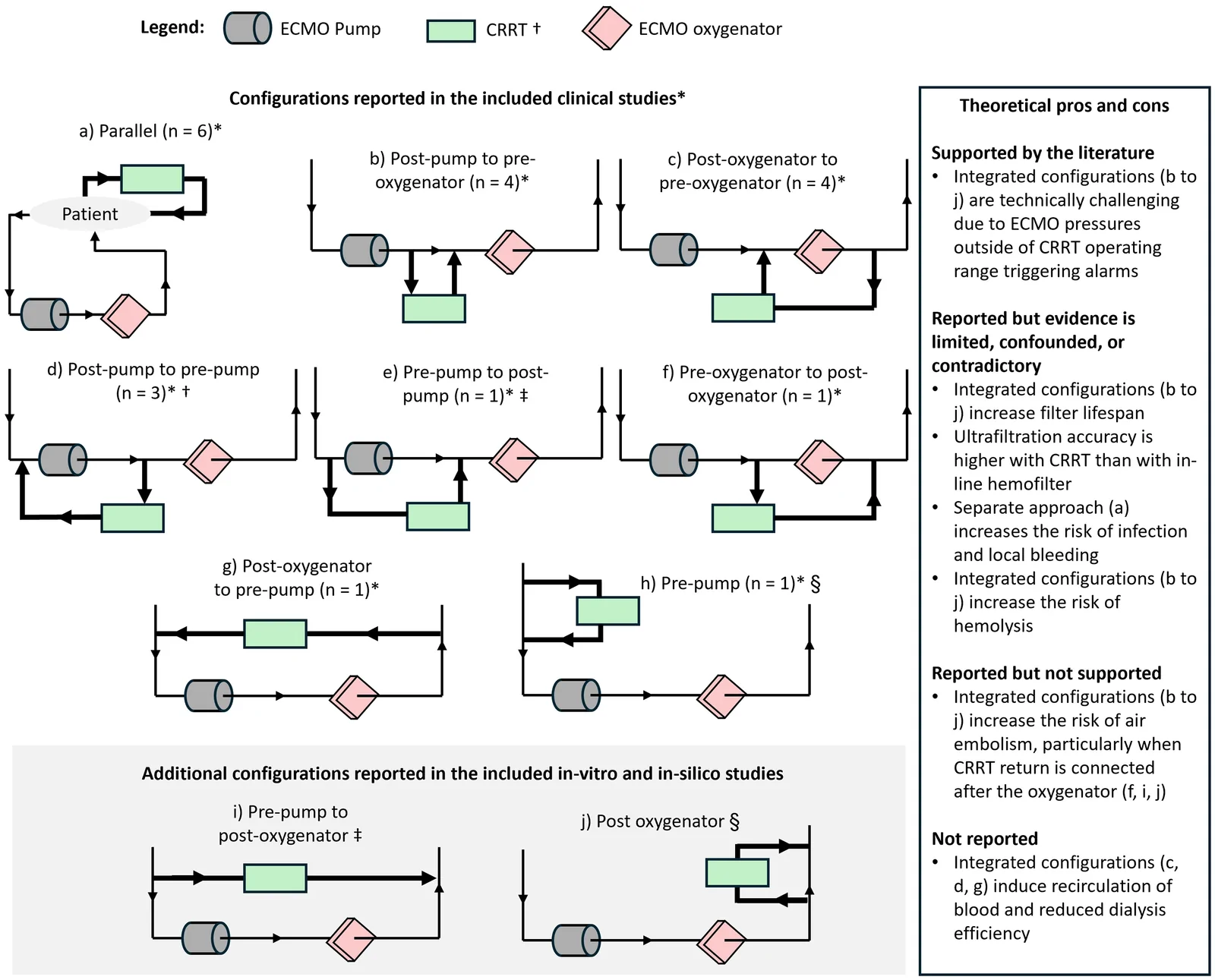
Configurations reported by included studies. Legend: Schematic representations of the configurations reported by the included clinical studies (top, a to h) and in-vitro or in-silico studies (bottom, i to j). Configurations are organized by the type of studies reporting them, the number of studies reporting them, and the similarities in the connections to the ECMO circuit. The arrows indicate the direction of the blood flow. Configurations always involve a CRRT device, except one involving an in-line hemofilter (†). * Refers only to the clinical studies included, without considering the number of in-vitro and in-silico studies reporting these configurations. All the configurations described in clinical studies have been investigated by at least one in-vitro or in-silico study. § indicates that the configuration was evaluated by an in-silico study, but no in-vitro study and ‡ indicates the opposite.

Except for the in-vitro and in-silico studies, the investigators compared no more than 3 configurations, with high variability in the integrated approaches considered.

Fig. 3 also summarizes the extent to which the theoretical advantages and disadvantages associated with the different ECMO-CRRT configurations are supported by the available literature. The evidence informing this categorization is presented in the following sections.

### Technical differences between various configurations and circuit lifespan

Additional file 3 summarizes the results from the included studies regarding technical differences, safety and efficacy of the different configurations. Overall, the main technical parameters affected by the configuration were pressures and hemofilter lifespan [16,17,[19], [20], [21],[23], [24], [25], [26], [27], [28], [29], [30]]. Other parameters, such as ECMO and RRT blood flow rates, were reported to be similar between the groups regardless of configuration [17,19,21,22,25,26].

In vitro and in silico studies showed that pressures within the CRRT circuit are strongly influenced by the choice of inlet and outlet connection sites when connected to the ECMO circuit and vary with the ECMO blood flow [29,30]. Consistently, clinical studies reporting pressures at different points of the ECMO and RRT circuits observed low pressures upstream of the ECMO pump [16,22]. Three other clinical studies proposed protocols and solutions for managing the high pressures in integrated CRRT circuit, including additional lines for pressure control and successful modification of the location of the inlet and outlet of the CRRT [16,17,23]. In their randomized controlled trial, Tangjitaree et al. (2026) reported the need to switch from the integrated post-pump → pre-oxygenator to parallel approach due to high pressure in one case, mentioning manageable pressures and interactions between the ECMO and CRRT devices [24].

Among the clinical studies comparing the parallel approach to an integrated one, Schönfelder et al. (2025) showed that pressures in the access, effluent or return lines were significantly lower in the parallel group compared to the integrated post-oxygenator → preoxygenator group (integrated: 110 [58–150] mmHg for the access line vs. parallel RRT device: −25 [−38–55] mmHg, *p < 0.01* and integrated: 170 [118–200] mmHg in the return line vs. parallel: 50 [23–138] mmHg, *p < 0.01*). The ECMO blood flow was not different between both groups (4.4 [3.6–5] L/min vs. 4.6 [3.7 − 5.8] L/min, *p = 0.29*), nor was the CRRT blood flow (100 [100 – 100] mL/min in both groups) [26]. This result was also confirmed by Worku et al. (2021) comparing 22 runs of RRT in the parallel configuration to 22 in the integrated post-pump → pre-oxygenator scheme with non-different ECMO and CRRT blood flow between the groups [19].

Despite these differences in circuit pressures, the impact of configuration on hemofilter lifespan remains uncertain. Wu et al. (2023) observed higher filter lifespan with the integrated approach (n = 70) compared to the parallel one (n = 30) (parallel: 12 ± 3 h vs. integrated: 47 ± 14 h; *p < 0.01*) [22] while Schönfelder et al. (2025) did not observe a difference in filter clotting events nor CRRT number of changes between the integrated (n = 57 patients) and parallel group (n = 48) (filter clotting: 30% vs. 25%; p *= 0.58*; circuit change 39% vs. 46%; p = *0.45*) [26]. More recently, Tangjitaree et al. (2026) conducted a randomized controlled trial showing that the CRRT filter lifespan (primary outcome) was not different between the parallel and integrated post-pump → pre-oxygenator groups (parallel, as treated: 70 [48–83] vs. 70 [48–97] h, *p = 0.39*) [24]. The discrepancies between these studies in terms of anticoagulation strategies, ECMO populations (V-A, V-V) and connection schemes for integrating the CRRT device into the ECMO circuit could explain the divergent findings reported by the authors. vs. vs. vs. As highlighted by the critical appraisal analysis presented in additional file 4, uncorrected biases emerged in other clinical studies reporting this outcome – such as differences in prescribed RRT dose between the comparison groups, substantial heterogeneity in anticoagulation strategies, and methodological flaws in analysis – precluded meaningful interpretation of results related to circuit configurations.

### Efficacy achieved depending on different connection schemes

Overall, no consistent difference in RRT efficacy, assessed through solute clearance (e.g., serum creatinine and blood urea nitrogen) and ultrafiltration efficiency, was observed between parallel and integrated CRRT configurations. Schönfelder et al. (2025) found no differences in efficacy between the parallel and integrated post-oxygenator → pre-oxygenator configurations despite similar ultrafiltration rate and dialysate flow set for the two groups. [26]. Similarly, Worku et al. compared the efficacy of the parallel and post-pump → pre-oxygenator approaches through 22 CRRT runs and did not find different ultrafiltration efficiency (ratio of actual vs. set ultrafiltration volume) between the two groups (91% for the parallel one vs. 88% for the integrated approach, *p = 0.78*) or decrease in serum creatinine (51% vs. 57%, *p = 0.57*) [19]. On the other hand, Ciullo, Knecht et al. (2023) found improved blood urea nitrogen clearance comparing the integrated approach (post-oxygenator → pre-oxygenator) to the parallel one, with significant decrease in blood urea nitrogen 12 h, 24 h and 48 h after the beginning of the treatment, (BUN decrease at 24 h (−5 [−12–0] mg/dL in integrated group vs. − 1 [−4–1] mg/dL in the parallel group; *p = 0.04*)). However, the authors did not report the prescribed CRRT dose, which may have biased these observations [21].

The only study comparing an integrated CRRT device with an in-line hemofilter demonstrated significantly greater ultrafiltration accuracy with CRRT in the pediatric population [15]. In the context of this study, accuracy refers to normalized ultrafiltration error defined as (physician specified fluid loss per 24-h period – actual fluid loss per 24-h period) / patient body weight (kg). Across several days of treatment, the authors observed a normalized CRRT error of 1.2 [−121–334] mL in the integrated CRRT group vs. normalized error of 13.1 [−151–2,188] mL in the in-line hemofilter group, *p < 0.001*).

### Comparison of different configurations regarding complications

The included clinical studies reported a broad range of complications, including thromboembolic events, bleeding, hemolysis, bloodstream infection, and air embolism, without identifying a consistently safer ECMO-RRT configuration. The additional vascular access required for the parallel approach was associated with increased catheter-site bleeding in one study (93%, 28/30 vs. 0), although the severity of these events was not reported and make it difficult to assess their clinical significance [22]. Schönfelder et al. (2025) highlighted an increased risk of thromboembolism related to the integration of CRRT into the ECMO circuit (4/57 (7%) vs. 0/48 (0%), *p = 0.06*) [26]. Alternatively, Raja et al. (2023) observed 3 cases of hemolysis in the integrated group, vs. 1 case of hemolysis, 1 case of air embolism and 1 case of heparin-induced thrombocytopenia in the parallel group of their 20-patient cohort. However, interpretation was limited by important differences in anticoagulation strategies between groups [20]. Finally, Tangjitaree et al. (2026) observed that the rate of complications such as hemolysis, exit site bleeding, systemic bleeding, blood transfusion, and new-positive blood culture was not different between the two groups compared [24].

### Impact of the configurations on mortality

In-hospital or ICU mortalities were reported by 9 studies, ranging from 20% in the study conducted by Raja et al. (2018) to 89% in those performed by Schönfelder et al. (2025).

Among the comparative studies, no significant difference in short-term mortality was observed between the parallel and integrated configurations, including post-oxygenator → pre-oxygenator [20,21,26] vs. and post-pump → pre-oxygenator approaches [24]. Biases related to the heterogeneous population of ECMO patients (V-V or V-A), and different or unreported characteristics of the patients at baseline in the other included clinical studies, preclude meaningful interpretation of the mortality depending on the configurations investigated.

### Beyond the research evidence: clinical practices

The integration of RRT and ECMO is highly heterogeneous among clinical studies and real-world practice, confirmed by the 4 surveys on medical practices included. Indeed, in the survey conducted by Thy et al. (2022) of 165 respondents, 61% used the ECMO circuit as the CRRT access while 39% preferred separate vascular access [34]. Similarly, Bidar et al. (2021) showed heterogeneous strategies for V-A ECMO, with 40% connecting RRT to the ECMO circuit, 28% using it in parallel, and 32% combining both approaches [31]. These proportions were similar for V-V ECMO. By contrast, these insights were very different for pediatric respondents with 80% declaring integrating the RRT into the ECMO circuit. Similar findings were published by Gorga et al. (2022) and Fleming et al. (2012), surveys with greater representation of pediatric specialists [32,33]. Gorga reported that 89% of participating centers used the ECMO circuit for RRT, with only one center relying solely on independent access and five combining both methods [33], and Fleming et al. (2021) showed that 21.5% used an in-line hemofilter and 50.8% integrated RRT into the ECMO circuit [32].

The survey conducted by Bidar et al. (2021) highlighted substantial variability in the integrated ECMO-RRT configurations used across centers [31]. Among respondents performing integrated ECMO-RRT, the post-pump → pre-oxygenator configuration was the most used (57%), followed by the pre-pump → pre-pump (18%), pre-pump → post-pump and post-oxygenator → pre-oxygenator (both 13%) configurations. Pressure alarms were encountered in more than 10% of sessions by 61 respondents, most frequently due to elevated access (39%) or return line (18%) pressures, and 32% reported switching to the parallel approach because of persistent alarms.

Similarly, Thy et al. (2022) reported that 33% of respondents had encountered at least one technical issue during integrated ECMO-RRT, including persistent pressure alarms, air embolism, or insufficient CRRT blood flow [34]. Among centers not performing integrated ECMO-RRT, the main barriers were the absence of institutional protocols (31/65), lack of guidelines (19/65), safety concerns (20/65), difficulties managing pressure alarms (12/65), and the absence of dedicated Y-connectors on the ECMO circuit (12/65). Among centers using integrated ECMO-RRT, safety considerations and pressure management were also identified as the principal factors guiding the choice of connection strategy (64% and 26% of respondents, respectively), although the survey did not distinguish the specific inlet and outlet connection sites.

## Discussion

Our scoping review highlights that, despite RRT being frequently indicated in ECMO patients, the current body of literature does not define the optimal configuration of the combined modalities. Previous technical reviews have described several theoretical advantages and drawbacks for the different ECMO-RRT configurations [5,6]. Parallel approaches are generally considered easier to implement because the two devices operate independently, but require additional vascular access, potentially increasing the risk of catheter-related bleeding, thrombosis, and infection. Conversely, integrated approaches eliminate the need for additional vascular access but expose the CRRT circuit to non-physiological pressures, complicating circuit management and raising concerns regarding pressure alarms and air embolism. However, our review shows that only a subset of these proposed advantages and drawbacks has been evaluated clinically. While pressure-related challenges in integrated configurations have been reported in comparative studies, the commonly perceived risk of air embolism is not clearly supported by the available literature [24,31,34]. Similarly, Thy et al. reported discrepancies between perceived and observed complications during integrated ECMO-RRT, with blood loss being cited as a concern by 10% of respondents despite only 2% reporting having encountered this complication in clinical practice [34].

More broadly, the available evidence remains limited, with a few comparative studies, limited sample sizes and high heterogeneity in both the circuit configurations described and the outcomes reported. Surveys on medical practices also revealed substantial variability in configurations used and anticoagulation strategies between ECMO centers. In addition, many studies were highly biased due to a heterogeneous population of patients (V-V, V-A and V-AV ECMO mixed) and unreported or uncorrected differences between the groups compared.

This heterogeneity precluded meaningful comparisons and made it difficult to derive standardized recommendations for clinical practice. In the absence of formal guidelines, medical practices are largely driven by local experience, individual concerns, technical preference, and institutional convention rather than robust scientific evidence, as highlighted by survey data [34]. Currently, Bidar et al. are conducting a randomized clinical trial (NCT06038162) comparing a parallel approach to an integrated one (precise configuration not described). Hemofilter lifespan is the primary outcome and clinical outcomes are secondary [35]. This study may provide valuable additional insights into the impact of integrating CRRT into the ECMO circuit for V-A ECMO patients and underscores the importance of future clinical studies that report not only the use of RRT but also the specific configuration employed.

Beyond the outcomes reported in this review, several important parameters remain unaddressed. For instance, priming volume is a critical issue in the pediatric population due to their small circulating blood volume, and it is higher when performing RRT in parallel to ECMO. Indeed, providing RRT and ECMO in parallel requires priming 2 separate extracorporeal circuits, substantially increasing the overall priming volume. Integration approaches enable shorter tubing and fewer pumps. Additionally, ECMO circuits do not always have connection ports, making it difficult for medical teams to perform the integrated approach [34]. Finally, the difference between ultrafiltration targets and final delivered dose under different configurations remains uncertain. Previous studies suggest it could be substantially affected when RRT is integrated into the ECMO circuit [36,37].

Exposure of the RRT device to highly positive or negative pressures as a result of connection to the ECMO circuit, can affect circuit performance and the reliability of pressure monitoring and derived parameters. This may lead to discrepancies between set and effective blood flow. Variations in inlet and outlet pressures also influence the measured transmembrane pressure, which can in turn affect filter performance, delivered dose, and ultrafiltration accuracy. In the survey by Thy et al. (2022), 5% of the respondents declared having encountered insufficient RRT blood flow when RRT is integrated into the ECMO circuit [34]. Therefore, it is essential to evaluate the differences between the therapeutic dose defined by the medical team, and the actual treatment dose delivered under the different configurations.

In addition to technical aspects, broader organizational and human factors are underexplored. Staff training and the implications for monitoring – including the burden of managing alarms and the effect on nursing workload and quality of care – are largely not addressed in the literature, although three of the studies included in our analysis aimed to propose solutions for managing pressures in the CRRT circuit [16,17,23]. In this context, implementation-focused technical notes, such as that by Bidar et al., provide practical guidance on ECMO-RRT connection strategies and pressure management and may facilitate the standardized and safe implementation of integrated ECMO-RRT [38].

Future studies should systematically investigate not only mortality but complication rates, quality of the therapy delivery in terms of both expected and delivered dose of dialysis and ultrafiltration, as well as technical challenges such as pressure alarms. Given the important number of integrated approaches to consider, a systematic comparison of all potential configurations not only from a hemodynamic perspective but also in terms of safety (air embolism, blood trauma, inflammation) and quality of the therapy delivery (RRT clearance, ultrafiltration achieved) is clearly required. This could be achieved through in-vitro and in-silico studies first, to inform design of clinical studies.

Clinical studies should systematically report the type of ECMO and RRT modalities, model, and the exact configuration for their concurrent use. Adequate and standardized definitions of the outcomes measured – particularly complications – would be highly valuable for future evidence synthesis. Finally, future clinical studies should carefully consider, and comprehensively report, potential confounding factors, including baseline severity, biomarker levels, anticoagulation strategies, and circuit pressures and flows, to minimize bias when comparing two or more configurations. A summary of these recommendations is presented in Table 2.

**Table 2:** Title: Summary of recommendations for future non-clinical and clinical research on ECMO-RRT configurations.

Study type	Subtype	Recommendations
All studies		Adopt standardized terminology and definitions for ECMO-CRRT configurations. Report VA-ECMO and VV-ECMO separately whenever possible. Evaluate outcomes beyond traditional clinical endpoints, including delivered vs. prescribed CRRT dose, filter lifespan, circuit downtime, alarm burden, nursing workload, and cost-effectiveness.
Non-clinical (in vitro, in silico)		Investigate outcomes beyond flows and pressures, including therapy performance, pharmacokinetics, and safety biomarkers (e.g., hemolysis, coagulation, inflammation). Whenever feasible, evaluate the range of ECMO-CRRT configurations used in clinical practice to facilitate translation of findings.
Clinical	All	Report ECMO-CRRT configuration(s) and technical modalities in sufficient detail (connection sites, pressures, flows, anticoagulation strategy, CRRT modality, etc.). Report complications using standardized and detailed definitions, particularly bleeding, infection, and circuit dysfunction.
	Observational	Include details about ECMO-RRT configurations into multicenter registries. Report on the clinical context that motivated the selected configuration (e.g., vascular access limitations, patient anatomy, ECMO cannulation strategy, local expertise, or institutional practice) Account for key confounders, including anticoagulation strategy, ECMO and CRRT flows, circuit pressures, illness severity, and relevant baseline biomarkers.
	Interventional	Anticipate crossover between study groups and the sequential use of different integrated approaches by predefining technical criteria for switching configurations and systematically reporting the timing and reasons for each crossover.

Finally, some research is exploring fully integrated RRT and extracorporeal respiratory support systems in a single extracorporeal platform [39]. Proof-of-concept studies have been conducted by Wiegmann et al. (2013), followed by Martins Costa et al. (2025), to combine the properties of ECMO and RRT into a single device, thereby promising to decrease priming volume and limit the need for additional vascular access or connection of two distinct circuits [40,41]. Such studies extend the conceptual boundaries of extracorporeal support, paving the way for next-generation hybrid systems capable of providing simultaneous respiratory and renal support with simplified circuit management and potentially improved patient outcomes.

### Strengths and limitations

This scoping review systematically mapped the available evidence on the techniques to simultaneously provide RRT and ECMO and identified key knowledge gaps. Strengths include a comprehensive search strategy and explicit inclusion/exclusion criteria, focusing on studies that compare multiple approaches and reported explicit circuit configurations.

However, some limitations must be acknowledged. Many potentially relevant articles were excluded because they only reported one configuration or failed to report the ECMO-RRT interface in sufficient detail. While this may have reduced the breadth of studies included, it ensured that our synthesis focused on evidence directly relevant to the research question. Another limitation is that the conclusions of this review are inherently constrained by the methodological quality and heterogeneity of the included studies, which precluded more definitive conclusions regarding the comparative effectiveness and safety of the different ECMO-RRT configurations. Although we note the existence of ongoing clinical trials, their results were not yet available at the time of this review, limiting our ability to incorporate prospective data that may soon address some of the identified gaps.

## Conclusion

The current evidence on ECMO-RRT integration is limited, heterogeneous, and insufficient to support standardized clinical recommendations. Ongoing clinical trials and future systematic, rigorous, comparative studies are essential to clarify which approach is optimal for patients and medical staff. Generating such evidence will be critical to harmonize practices across centers and to inform guideline development in this evolving field.

## Authors’ contribution

Eden Kharrouba: conceptualization, methodology, investigation, visualization, writing (original draft)

Quentin Moyon: conceptualization, methodology, investigation, writing (review and editing)

Matthew Cove: writing (review and editing)

Jutta Arens: writing (review and editing)

Ana Martins Costa: writing (review and editing)

John F Fraser: writing (review and editing)

Jacky Y Suen: conceptualization, supervision, writing (review and editing).

## Consent for publication

Not applicable.

## Ethics approval and consent to participate

Not applicable.

## Funding

None.

## Competing interest

The authors declare that they have no competing interests.

## Availability of data and materials

All data generated or analyzed during this study are included in this published article [and its supplementary information files].
