# Supplementary material for: Comparison of the approaches to combine Renal Replacement Therapy with Extracorporeal Membrane Oxygenation – a Scoping Review
Source: Ann Intensive Care. 2026 Sep 21;16:100165. doi: 10.1016/j.aicoj.2026.100165 (PMC13656946; doi:10.1016/j.aicoj.2026.100165)
Supplement: Supplementary file 2 [file mmc2.docx]

# Additional file 1 – Queries used to retrieve the included studies

## Pubmed

( ECMO[tiab] OR "Extracorporeal Membrane Oxygenation"[tiab] OR ECLS[tiab] OR "Extracorporeal Life Support"[tiab] OR "Extracorporeal Circulatory Support"[tiab] OR "Extracorporeal Lung*"[tiab] OR "Extracorporeal Cardiopulmonary Resuscitation"[tiab] OR "mechanical circulatory support"[tiab] OR "extracorporeal support"[tiab] )

AND

( CRRT[tiab] OR CKRT[tiab] OR RRT[tiab] OR "Continuous Renal Replacement Therapy"[tiab] OR "Continuous Kidney Replacement Therapy"[tiab] OR "Renal Replacement Therapy"[tiab] OR "Kidney* Support"[tiab] OR "Kidney*"[tiab] OR "Renal Support"[tiab] OR "Kidney Replacement Therapy"[tiab] OR Hemofiltration[tiab] OR hemodiafiltration[tiab] OR CVVH[tiab] OR CVVHD[tiab] OR CVVHDF[tiab] OR Hemodialysis[tiab] OR "Dialytic Support"[tiab] OR dialysis[tiab] OR "extracorporeal renal support"[tiab] )

## Web of Science

TS=(ECMO OR "Extracorporeal Membrane Oxygenation" OR ECLS OR "Extracorporeal Life Support" OR "Extracorporeal Circulatory Support" OR "Extracorporeal Cardiopulmonary Resuscitation" OR "Extracorporeal Lung*" OR "mechanical circulatory support" OR "extracorporeal support") AND TI= (CRRT OR cart OR RRT OR "Continuous Renal Replacement Therapy" OR "Continuous Kidney Replacement Therapy" OR "Renal Replacement Therapy" OR "Kidney Replacement Therapy" OR "Kidney* Support" OR Kidney* OR "Renal Support" OR Hemofiltration OR Hemodiafiltration OR CVVH OR cgvhd OR cgvhd OR Hemodialysis OR "Dialytic Support" OR Dialysis OR "extracorporeal renal support")

## Embase

('extracorporeal membrane oxygenation'/exp OR 'extracorporeal membrane oxygenation' OR ECMO OR ECLS OR "extracorporeal life support") AND ('renal replacement therapy'/exp OR 'continuous renal replacement therapy' OR CRRT OR CVVH OR CVVHD OR CVVHDF OR hemofiltration OR hemodiafiltration OR dialysis) AND (connection* OR configur* OR interface* OR circuit* OR setup OR position* OR pre-pump OR post-pump OR pre-oxygenator OR post-oxygenator OR inline OR independent OR "in series" OR "in parallel") AND ([article]/lim OR [article in press]/lim) NOT (animal/exp NOT human/exp)

## Cochrane library

(ECMO OR "Extracorporeal Membrane Oxygenation" OR ECLS OR "Extracorporeal Life Support" OR "Extracorporeal Circulatory Support" OR "Extracorporeal Cardiopulmonary Resuscitation" OR "Extracorporeal Lung" OR "mechanical circulatory support" OR "extracorporeal support") AND (CRRT OR CKRT OR RRT OR "Continuous Renal Replacement Therapy" OR "Continuous Kidney Replacement Therapy" OR "Renal Replacement Therapy" OR "Kidney Replacement Therapy" OR "Kidney Support" OR Kidney OR "Renal Support" OR Hemofiltration OR Hemodiafiltration OR CVVH OR CVVHD OR CVVHDF OR Hemodialysis OR "Dialytic Support" OR Dialysis OR "extracorporeal renal support") in Title Abstract Keyword

## Clinicaltrials.gov

("ECMO" OR "Extracorporeal Membrane Oxygenation" OR "ECLS" OR "Extracorporeal Life Support" OR "Extracorporeal Circulatory Support" OR "Extracorporeal Cardiopulmonary Resuscitation" OR "Extracorporeal Lung" OR "mechanical circulatory support" OR "extracorporeal support") AND ("CRRT" OR "CKRT" OR "RRT" OR "Continuous Renal Replacement Therapy" OR "Continuous Kidney Replacement Therapy" OR "Renal Replacement Therapy" OR "Kidney Replacement Therapy" OR "Kidney Support" OR "Renal Support" OR "Hemofiltration" OR "Hemodiafiltration" OR "CVVH" OR "CVVHD" OR "CVVHDF" OR "Hemodialysis" OR "Dialytic Support" OR "Dialysis" OR "extracorporeal renal support")
