# Supplementary material for: Comparison of the approaches to combine Renal Replacement Therapy with Extracorporeal Membrane Oxygenation – a Scoping Review
Source: Ann Intensive Care. 2026 Sep 21;16:100165. doi: 10.1016/j.aicoj.2026.100165 (PMC13656946; doi:10.1016/j.aicoj.2026.100165)
Supplement: Supplementary file 3 [file mmc3.docx]

# Additional file 2 – Key findings of the included preclinical and clinical studies

| **Study** | **Country** | **Study type** | **Population** | **Key findings** |
| --- | --- | --- | --- | --- |
| **Symons et al. (2013)** | USA | Clinical, retrospective, single centre | Pediatrics (n = 42)  V-A/V-V: NA  In-line hemofilter post‑pump→pre‑pump (n = 30) vs integrated RRT device post‑pump→pre‑oxygenator or post‑pump→pre‑pump (n = 12)  In-line hemofilter group had higher initial creatinine (0.5 [0.1 – 4.8] mg/dL vs 0.4 [0.2 – 2.1] mg/dL; *p*<0.01) | - ICU/hospital LOS and in‑hospital mortality **non different** but mortality was high (ICU LOS: 33.5 [5 – 401] days for integrated CRRT vs 52 [11 – 139] days for in-line hemofilter, p = 0.44; Hospital LOS: 34 [5 – 401] days for integrated CRRT vs 70 [11 – 176] days for in-line hemofilter, p = 0.47; In-hospital mortality: 84% vs 79%, *p = 0.25*). - Integrated CRRT more accurate for ultrafiltration than in-line hemofilter (normalized error 1.2 [−121 – 334] mL vs 13.1 [−151 – 2,188] mL; *p < 0.001*). - Integrated CRRT was associated with shorter duration of ECMO (384 h vs 583 h, *p < 0.001*) and RRT (185 h vs 477 h, *p < 0.001*) than in-line hemofilter patients. |
| **De Tymowski et al. (2017)** | France | Clinical, single centre | Adults (n = 12)  V-A (n = 8); V-V (n = 4)  Integrated RRT device (n = 12); sequential: post‑pump→pre‑oxygenator, pre‑pump→post‑pump, pre‑pump→pre‑pump | Going from a post pump inlet connection to a pre pump  inlet enabled management of pressure triggered alarms. |
| **Na et al. (2018)** | South Korea | Clinical, single centre | Adults (n = 170)  V-A (n = 127); V-V (n = 40);  Mixed (n = 3)  Integrated RRT device post‑pump→pre‑pump with pressure lines: 0 (n = 16), 1 (n = 36), 2 (n = 118) | - Lower in-hospital mortality in the group of patients managed with double line pressure control (69% for no line vs 75% for single line vs 48% for double line, *p = 0.01*), although longer duration of ECMO support (80 [53 – 467] h for no line vs 93 [32 – 189] h for single line vs 184 [93 – 501] h for double line, *p = 0.003*) - Complication rates non different - Similar safety based on the measured variations of the biomarkers - Increased CRRT circuit lifespan for the double line group (21.8 [11.6 – 31.8] h for no line vs 23.0 [15.0 – 34.6] h in single-line vs 45.0 [29.0 – 63.7] h for double line, *p < 0.001*) |
| **Gorga et al. (2020)** | North America | Clinical, retrospective observational, multicentric | Pediatrics (n = 357)  V-A (n = 265); V-V (n = 92)  Integrated RRT device (n = 81) vs in-line hemofilter (n = 276)  connections not described | Lower in hospital mortality for integrated CRRT (42% vs  60%; *p = 0.005*). |
| **Worku et al. (2021)** | USA | Clinical, retrospective, single centre | Adults (n = 25)  V-A  Parallel (n = 22 runs) vs integrated RRT device post‑pump→pre‑oxygenator (n = 22 runs)  No record of inlet pressure in integrated RRT device setting to prevent constant alarms | - Creatinine decrease non different (51% integrated vs 57% parallel; *p = 0.57*) and UF precision non different (91% vs 88%; *p = 0.78*). - Number of RRT circuit changes non different (4.4 vs 3.8; *p = 0.54*). - Outlet pressure and effluent pressure significantly higher in parallel CRRT (122 mmHg and 137 mmHg vs 293 mmHg and 321 mmHg, respectively, *p > 0.01*). - 2 patients needed to be converted from integrated to parallel because of low RRT flow and circuit clotting |
| **Liu et al. (2021)** | China | Clinical, single centre | Adults (n = 21)  V-A (n = 16); V-V (n = 3); V-AV (n = 2)  Integrated RRT device post‑pump→pre‑pump (n = 8) vs post‑oxygenator→pre‑oxygenator (n = 13) | - Comparable groups in terms of ECMO and CRRT blood flows, homeostasis time, ECMO + CRRT duration - ICU length of stay and prognosis non different - Comparable results related to the biomarkers measured both regarding safety and efficiency of the therapies - Non different fluid balance group - CRRT filter lifespan non different |
| **Wu et al. (2023)** | China | Clinical, retrospective observational, single centre | Adults (n = 100)  V-A (n = 70); V-V (n = 30)  Parallel (n = 30) vs integrated RRT device post‑oxygenator→pre‑oxygenator (n = 45) or post‑oxygenator→pre‑pump (n = 25) | - Comparable groups in terms of ECMO running time and blood flow - Discharge survival rate non different (46.7% for parallel vs 40.0% for integrated RRT device, *p = 0.536*) but local bleeding observed in 93% of the patients in the parallel group - Reduced CRRT initiating time for the integrated group (parallel: 49.0 ± 12.1 min vs integrated: 14.6 ± 2.1 min, *p < 0.001*) and increased filter lifespan (parallel: 11.5 ± 3.2 h vs integrated: 47.3 ± 14.0 h, *p < 0.001*) with equally reached therapeutic dose   High pressure in CRRT return line can be managed by changing the position from post-pump and pre-oxygenator to pre-pump |
| **Raja et al. (2023)** | United Kingdom | Clinical, single centre,  prospective | Adults (n = 20)  V-V  Parallel (n = 10) vs integrated RRT device post‑oxygenator→pre‑oxygenator (n = 10) | - In-hospital mortality was non different between the integrated and parallel groups (20%) but ECMO duration was higher in the integrated one (integrated: 25 days vs parallel: 13.5 days) - 3 patients experienced hemolysis in the integrated group (post to pre-oxygenator) while in the parallel group, 1 patient experienced air embolism, 1 experienced hemolysis, and 1 had heparin-induced thrombocytopenia - ECMO circuit changes were equal in both groups of patients - Average filter lifespan and filter runs completing 72 hours were higher in the integrated group (integrated: 42 h of filter lifespan vs parallel: 28 h in the parallel group and 40% of the filter runs completing 72 h vs 13.8% in the parallel group) |
| **Ciullo, Knecht et al. (2023)** | USA | Clinical, retrospective observational, single centre | Adults (n = 33)  Type of ECMO: NA  Parallel (n = 26) vs integrated RRT device post‑pump→pre‑oxygenator (n = 7) | - ICU mortality and length of stay non different (respectively, 4 (67%) in the integrated group vs 17 (63%), *p = 0.21* and [14 – 29] days for integrated group vs 22 [15 – 51] days for parallel group, *p = 0.695*) - BUN decreased more with integrated RRT device at 24 h (−5 [−12 – 0] mg/dL vs -1 [−4 – 1] mg/dL*;* p = *0.04*). - Platelets higher at 72 h with integrated RRT device (94 k/µL vs 71 k/µL; p = *0.008*) |
| **Huang et al. (2023)** | China | Clinical, single centre | Adults (n = 120)  V-A (n = 60); V-V (n = 60)  Integrated RRT device (n = 120); pre‑oxygenator→post‑oxygenator with additional pressure controls (n = 60) vs without (n = 60) | - Adding additional control lines does not alter the efficiency of the CRRT therapy based on the evolution of creatinine and blood urea nitrogen evolution during the treatment - The group with additional pressure control benefitted from a longer filter lifespan (38.24 ± 4.12 h vs 27.12 ± 5.13 h, *p < 0.01*) and higher CRRT loop pressure (45.15 ± 1.74 mmHg vs −69.00 ± 2.12 mmHg, *p < 0.001*) |
| **Schönfelder et al. (2025)** | Germany | Clinical, retrospective observational, single centre | Adults (n = 105)  V-V  Parallel (n = 48) vs integrated RRT device post‑oxygenator→pre‑oxygenator (n = 57)  Comparable baseline labs, CRRT & ECMO settings | - Safety outcomes non different (mortality, ICU LOS, days alive, AKI recovery, bacteremia/CVC infections) - Trend to more thromboembolism with integrated RRT device (4 (7%) vs 0; p = 0.06). - Equal RRT efficiency regarding serum creatinine - Filter clotting and CRRT circuit change within 72 h non different (30% vs 25%; *p = 0.58*; circuit change 39% vs 46%; *p = 0.45*) |
| **Tangjitaree et al. (2026)** | Thailand | Clinical, randomized control trial, multicenter | Adults (n = 80)  V-A (n = 67); V-V (n = 13)  Parallel (n = 40) vs integrated RRT device post‑pump→pre‑oxygenator (n = 40)  Patients randomized per blocks of 4; 9 patients treated with the parallel approach switched to integrated approach and 1 patient receiving integrated approach switched to parallel one (cross-over) | - Primary outcome: adjusted and non-adjusted CRRT filter lifespan not different between the two groups by intention to treat nor as treated (as treated unadjusted circuit lifespan, integrated: 70 [48 – 97] vs parallel: 70 [44 – 83] h, *p = 0.39*) - Secondary outcomes: non different number of CRRT machine alarm, high access and return pressure alarm, low access and return pressure alarm, transmembrane pressure alarm 28-day mortality non-different (parallel: 35% (n = 14) vs integrated 32.5% (n = 13), *p = 0.81*)  Serious adverse events such as exit site bleeding, systemic bleeding, hemolysis, blood transfusion, new-positive blood culture non different No air embolism reported for both groups |
| **Suga et al. (2017)** | Japan | In‑vitro | V-V and V-A  Integrated RRT device; post‑pump→pre‑pump (V-V) and post‑oxygenator→pre‑pump (V-A) | - Pressures before the pump are lower than between the pump and the oxygenator or post-oxygenator - Intra-circuit pressures vary greatly with the ECMO flow   Inlet and outlet pressures of the hemofilter increases with CRRT flow |
| **Profeta et al. (2018)** | USA | In‑vitro | Neonatal ECMO circuit  V-A (Centrifugal or roller pump with Quadrox-ID pediatric oxygenator)  Integrated RRT device; 7 integrated configurations | Compared to an ECLS circuit without CRRT:   - Post‑pump→pre‑oxygenator and post‑pump→post‑oxygenator **non different** overall hemodynamics, except higher pressure drops for post‑pump→post‑oxygenator. - Returning pre‑pump tends to ↓ arterial cannula flow and mean pressures pre‑oxygenator/post‑arterial cannula; ↓ total pressure drop & total hemodynamic energy; but post‑oxygenator→pre‑pump ↑ total hemodynamic energy loss.Pre‑pump→post‑oxygenator, pre‑pump→post‑pump, post‑oxygenator→pre‑oxygenator generally ↑ pre‑oxygenator/arterial flows & mean pressures, ↑ pressure drops & total hemodynamic energy (except post‑oxygenator→pre‑oxygenator). - Effects depend on ECMO flow; some findings invert at low flow (200 mL/min). |
| **Shank et al. (2018)** | USA | In‑vitro | Neonate  V-A  Integrated RRT device; post‑pump→pre‑oxygenator, pre‑pump→post‑pump, post‑pump→pre‑pump | - The position of CRRT had no impact on overall hemodynamics in extracorporeal life support (ECLS) circuits with centrifugal pumps. However, in circuits with roller pumps, positioning significantly altered hemodynamic performance. - Connecting CRRT between post-pump to pre-oxygenator maintained stable hemodynamic parameters regardless of pump type. Connecting the CRRT pre-pump to post-pump increased flow, circuit pressures, and energy transmission in roller pump circuits - Recirculating blood through the ECLS pump with CRRT connected post-pump to pre-pump led to reduced flow and energy delivery in roller pump circuits.   In roller pump systems, connections post-pump to pre-oxygenator and post-pump to pre-pump yielded lower microemboli counts and volumes than pre-pump to post-pump |
| **Thiel et al. (2025)** | Germany | In‑silico | Adults (n = 8)  V-A  Integrated RRT device; 9 integrated configurations | Differences exist in pressures and right ventricle volume during a cardiac cycle among the 9 configurations investigated, with lower pressures observed for pre-pump connection.  These pressures in access and return lines are greatly affected by ECMO pump flow |

**Caption: Comprehensive report of the key findings of the clinical and preclinical studies included. These results should be carefully considered regarding the critical appraisal provided previously in Table S1 as important biases in some studies preclude meaningful interpretation of their results.**

*Abbreviations: ECMO, extracorporeal membrane oxygenation; CRRT, continuous renal replacement therapy; V-A, veno-arterial; V-V, veno-venous; V-AV, veno-arterial-venous; CVVH, continuous veno-venous hemofiltration; CVVHD, continuous veno-venous hemodialysis; CVVHDF, continuous veno-venous hemodiafiltration; SCUF, slow continuous ultrafiltration; IHD, intermittent hemodialysis; SLED, sustained low-efficiency dialysis; ICU, intensive care unit; LOS, length of stay; NA, not available*
