# Supplementary material for: Comparison of the approaches to combine Renal Replacement Therapy with Extracorporeal Membrane Oxygenation – a Scoping Review
Source: Ann Intensive Care. 2026 Sep 21;16:100165. doi: 10.1016/j.aicoj.2026.100165 (PMC13656946; doi:10.1016/j.aicoj.2026.100165)
Supplement: Supplementary file 4 [file mmc4.docx]

# Additional file 3 – Critical Appraisal of the 11 non-randomized clinical studies included

**1A) Detail of the items constituting the JBI checklist for cohort studies – specific criteria considered in the context of this scoping review are listed below each item**

1. **Were the two groups similar and recruited from the same population?**
   – Comparable illness severity indicators
   – Same ECMO mode
2. **Were the exposures measured similarly to assign participants to both exposed and unexposed groups?**
   – Treatment allocation predefined and changes in allocation during the course of study adequately reported
   – Similar ECMO and CRRT circuit
   – Same timing of intervention
3. **Was the exposure measured in a valid and reliable way?**
   – Clear and detailed configuration description
4. **Were confounding factors identified?**
   – Severity/confounders reported (e.g., SOFA, APACHE, lactate)
   – Mortality confounded by severity
   – Filter clotting confounded by anticoagulation
   – Hemolysis confounded by anticoagulation and circuit pressures
   – Fluid balance correction influenced by flow
   – Serum electrolytes influenced by flow
5. **Were strategies to deal with confounding factors stated?**
   – Propensity score matching
   – Adjusted Cox, linear, or logistic regression models
6. **Was the cohort free of outcomes at baseline?**
7. **Were outcomes measured in a valid and reliable way?**
8. **Was follow-up time sufficient for outcomes to occur?**
9. **Was follow-up complete?**
10. **Were strategies used to address incomplete follow-up (if present)?**
11. **Was appropriate statistical analysis used?**
    – Analytical approach appropriate to study type and data

**1B) Table summarizing the critical appraisal conducted**

| Study | Population | Exposures | | Confounding factors | | Outcomes | | Follow-up | | | Statistical analysis |
| --- | --- | --- | --- | --- | --- | --- | --- | --- | --- | --- | --- |
|  | Item 1 | Item 2 | Item 3 | Item 4 | Item 5 | Item 6 | Item 7 | Item 8 | Item 9 | Item 10 | Item 11 |
| Symons et al. (2013) | N | Y | Y | Y | Y | Y | Y | NA | | | Y |
| De Tymowski et al. (2017) | Y | Y | Y | NA | NA | Y | Y |  |  |  | NA |
| Na et al. (2018) | N | Y | Y | Y | N | Y | Y |  |  |  | Y |
| Gorga et al. (2020) | CT | Y | CT | N | N | Y | Y |  |  |  | NA |
| Worku et al. (2021) | CT | CT | Y | Y | Y | Y | Y |  |  |  | CT |
| Liu et al. (2021) | N | Y | Y | N | N | Y | N |  |  |  | Y |
| Wu et al. (2023) | N | Y | Y | CT | CT | Y | CT |  |  |  | Y |
| Raja et al. (2023) | Y | CT | Y | Y | N | Y | Y |  |  |  | NA |
| Ciullo, Knecht et al. (2023) | CT | CT | N | Y | Y | Y | Y |  |  |  | Y |
| Huang et al. (2023) | N | N | N | N | N | Y | CT |  |  |  | N |
| Schönfelder et al. (2025) | Y | CT | Y | Y | Y | Y | Y |  |  |  | Y |

**Caption: JBI critical appraisal decision for clinical, cohort studies. “Y/Yes” means that the criterion was met or adequately addressed according to the JBI Checklist for Cohort Studies. As the aim of the different studies varied and as various outcomes of interest were often reported by each study, answers for each item reflect an overall judgement.**

*Abbreviations: Y, Yes: N, No; CT, can’t tell or unclear; NA, non-applicable*
